# The Significance of Vertigo

**Published:** 1927

**Authors:** E. Watson-Williams


### THE SIGNIFICANCE OF VERTIGO.

BY

E. WATSON-WILLIAMS, M.C., CH.M., F.R.C.S. Ed.

"VERTIGO," says Nicholas Culpeper, "is a disease wherein a man thinketh all that he sees turns round." The term is strictly applied only to symptoms which involve a sensation of turning, but is frequently employed to cover all varieties of giddiness. Hughlings Jackson defines it as "consciousness of the effect on motor centres of want of harmony between afferent impressions." We may be conscious of the want of harmony without vertigo: only when equilibrium is affected do we experience that sensation. Under the influence of appropriate stimuli, especially certain movements, vertigo is normal, and the absence of it pathological. We are now concerned with pathological vertigo, occurring apart from an adequate cause. It is unfortunate that one word has to do duty for so many varieties of sensation : the feelings evoked by rectilinear movement, by falling, swaying or jogging the body, are essentially quite distinct from the very characteristic sequelæ of turning, and a good deal of confusion exists in medical literature from the varied usage of vertigo to express here the latter sensation only, there the former also.

We may recognise, then, two broad classes of giddiness :—

(*a*) Dizziness, swimming in the head, a vague sense of impaired equilibrium. Feelings that the patient or his surroundings, including perhaps the ground, sways to and fro or up and down. Sensations of falling.

(*b*) The sensation that the patient or his surroundings or both are turning round and round. The latter is the classical or rotatory vertigo, a phrase almost exactly equivalent to "turning round."

Any variety of vertigo may be, and very commonly is, due to disease of the ear. Where the symptom is either severe or persistent, this possibility is not likely to be overlooked, especially if there is deafness or otorrhœa to assist the diagnosis. In their minor degrees the general vertiginous sensations, though perhaps distressing, are usually vague and ill appreciated. They do not in themselves point to the implication of one part more than another: we may say they are "symmetrical." Apart from such serious conditions as diseases of the heart or vessels, profound alterations in the blood, or nervous disorder, we know they may be due to exhaustion, endogenous poisons, drugs, and even, it is claimed, to endocrine disturbance. Some assert that the vertigo in such cases is due to action on the labyrinth, but it seems equally possible that often the effect is direct on the cerebral cortex. At least these general conditions should affect both labyrinths equally; nor is there anything in the nature of these symmetrical vertiginous sensations themselves to suggest that this is not the case.

Vertigo of any variety occurring where there is other evidence of nervous disease, such as paralysis (including ocular palsies) or fits, is a matter for the neurologist. In both functional and organic nervous disease dizziness appears to be common. As regards the latter, a definite rotatory vertigo is suggestive of a localised disorder. In functional disorders, while there seems general agreement that true vertigo is not a symptom of hysteria, there is a conflict of views as to whether it may be due to neurasthenia. One may remark in this connection that a neurosis is a regrettably common sequela of true aural vertigo, and I am convinced that genuine neurasthenia may be due to the same cause. In any event, the significance of the symptom in functional nervous disease is sufficient to call for an examination of the ears. But we are concerned now with vertigo where it is the sole, or only prominent, evidence of any nervous lesion, and where in consequence there might be a risk of ascribing it to general disorders or even to imagination.

Though the general vertiginous sensations do not in themselves bear any clear indication of their origin, where there is or has been a true rotatory vertigo the position is different. This symptom can only occur from some alteration in the vestibular impulses reaching the brain. And in the absence of other evidence of nervous disease it is pathognomonic of disease of the ear. Nearly always the seeming rotation is in a transverse plane, that is, in the plane of the base of the skull, and in this case there must be something affecting the two labyrinths unequally. The sensation of turning is concise and definite. Months or years after even a slight attack the patient's description is clear and vivid : many even cannot refrain from illustrating the sensation at each account by a turning movement of the hand. Not only do the intrinsic qualities of the symptom impress the patient, but its traditional and well-founded association with apoplexy frequently terrifies. When Ménière in 1861 set out to describe the syndrome that bears his name, he chose as a title " Observations des maladies de l'oreille characterisèes par des symptomes de congestion cérébrale apoplectiforme." And so deeply rooted has been the suggestion of apoplexy that it has often been assumed that he wrote of a hemorrhage into the labyrinth. The wording of his descriptions of his famous case, such phrases as "une lymphe plastique remplaçant la liquide de Cotugno," will not bear this construction. Hemorrhage into the labyrinth appears to occur exclusively and rarely in leukæmia, and we have no evidence that Ménière ever observed it.

The seventeenth-century physicians were well aware of the association of vertigo with deafness and tinnitus. There are even passages in Hippocrates in which vertigo in otitis is mentioned as a presage of disaster. The importance of Ménière's observations lay in the demonstration of an actual labyrinthine lesion producing vertigo, and it has rather overshadowed our conceptions on the subject. Actual intra-labyrinthine disease even from suppuration or otosclerosis is relatively infrequent. And at least the victims have this compensation, that the vertigo disappears following the destruction of the labyrinth itself. By far the most frequent cause of vertigo is catarrhal otitis. The attacks are seldom so severe, though the most extreme degrees of giddiness sometimes occur in this disease. But the labyrinth may remain for years alive and sensitive, and the resulting vertigo become a source of protracted misery.

When vertigo occurs with deafness or tinnitus, it is not likely to present difficulty. But for two reasons these other aural symptoms may be absent. In the first place, the subject of unilateral deafness is frequently oblivious of it ; secondly, vertigo may precede by a long interval all other evidence of aural disease. The giddiness in such patients is usually slight, and often vague ; but if there have been any attacks, albeit brief and transient, of true rotatory vertigo, it is certain that intracranial disease or some affection of the ear is responsible.

It was at one time proper to distinguish *vertigo ab auri læsa* from *vertigo a stomacho læso.* The idea dies hard that gastric disturbance can cause a true rotatory vertigo. The patient comes for advice for his " biliousness," nausea and vomiting being the conspicuous symptoms. He may omit to mention any vertigo, regarding it as a symptom of biliousness; or the vertigo may have been present long before the symptoms that are worrying him. He may even scout the idea that an ear which has been " hard of hearing " all his life has anything to do with his recent illness. But though vestibular disturbance can and regularly does cause nausea, and even vomiting, it is doubtful whether a gastric disorder ever produces a true rotatory vertigo. Over thirty years ago Gowers wrote, " I do not think it is certain that there is such a thing as definite vertigo of purely gastric origin. In 90 per cent. of all cases of definite giddiness a morbid state of the labyrinth is the real cause."

Very frequently one sees patients on account of deafness, whose aural disease has not even been suspected of any connection with the " bilious attacks " or " gastritis " of preceding years. Yet there has been all the time, in the form of a definite story of rotatory vertigo, a clear indication of vestibular disorder. It is important, since the gastric symptoms are readily amenable to aural treatment, especially in the early phase. In this connection I wish to bring to your notice an observation that I believe to be new. The nausea of vestibular origin is commonly at its worst on rising. The patient therefore is slightly nauseated for the first two or three hours of the day, even though the effect passes off later, and in consequence he has no appetite for breakfast. Even those whose vertigo as such is trivial may show this " breakfast sign," which I have never found absent in a subject of definite labyrinthine vertigo. The patient may be well nourished, and assert that the appetite is good—he can "eat anything." But on enquiry as to the meals actually taken he will say, "Well, for breakfast I have tea and a little toast, I never want anything more." Later on the appetite is quite good. I do not wish to assert that labyrinthine vertigo is the only cause of this morning anorexia, which would be manifestly untrue. But if a patient, whose appetite is otherwise good, cannot regularly "face his breakfast" the condition of the ears should excite interest.

One of the otologist's troubles is that his cases are "selected." Nearly all the patients come on account of some definite aural trouble. He is therefore compelled to rely upon the opinions of others as to the incidence generally of symptoms that he may observe not immediately indicative of disease of the ears. It is because the history in so many cases reveals evidence of the existence of aural disease, preceding by months or years the deafness or tinnitus for which advice is sought, that I venture to emphasise the significance of true rotatory vertigo.

### SUMMARY.

1. Rotatory vertigo, in the absence of other signs of nervous disease, is evidence of an aural affection.

2. Vertigo may be the first sign of disease of the ear.

3. Alimentary disorders do not by themselves cause rotatory vertigo.

4. The breakfast sign: nausea or anorexia confined to the first part of the day may be of aural origin.

